# Cascaded Tuning to Amplitude Modulation for Natural Sound Recognition

**DOI:** 10.1523/JNEUROSCI.2914-18.2019

**Published:** 2019-07-10

**Authors:** Takuya Koumura, Hiroki Terashima, Shigeto Furukawa

**Affiliations:** NTT Communication Science Laboratories, Atsugi, Kanagawa, Japan 243-0198

**Keywords:** amplitude modulation, deep neural network, neural tuning, single-unit recording

## Abstract

The auditory system converts the physical properties of a sound waveform to neural activities and processes them for recognition. During the process, the tuning to amplitude modulation (AM) is successively transformed by a cascade of brain regions. To test the functional significance of the AM tuning, we conducted single-unit recording in a deep neural network (DNN) trained for natural sound recognition. We calculated the AM representation in the DNN and quantitatively compared it with those reported in previous neurophysiological studies. We found that an auditory-system-like AM tuning emerges in the optimized DNN. Better-recognizing models showed greater similarity to the auditory system. We isolated the factors forming the AM representation in the different brain regions. Because the model was not designed to reproduce any anatomical or physiological properties of the auditory system other than the cascading architecture, the observed similarity suggests that the AM tuning in the auditory system might also be an emergent property for natural sound recognition during evolution and development.

**SIGNIFICANCE STATEMENT** This study suggests that neural tuning to amplitude modulation may be a consequence of the auditory system evolving for natural sound recognition. We modeled the function of the entire auditory system; that is, recognizing sounds from raw waveforms with as few anatomical or physiological assumptions as possible. We analyzed the model using single-unit recording, which enabled a fair comparison with neurophysiological data with as few methodological biases as possible. Interestingly, our results imply that frequency decomposition in the inner ear might not be necessary for processing amplitude modulation. This implication could not have been obtained if we had used a model that assumes frequency decomposition.

## Introduction

**Figure 1.**
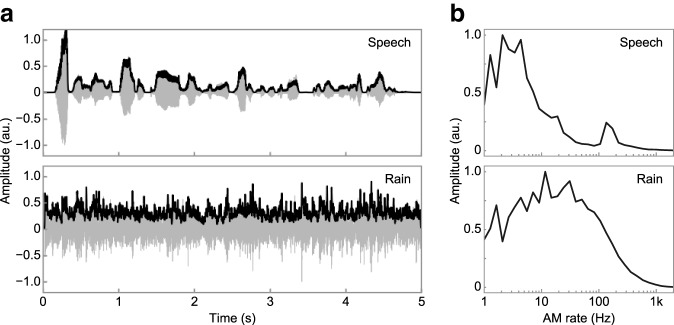
Rich repertoires of AM in natural sounds. ***a***, Examples of sound waveforms (gray) and their amplitude envelopes (black) of natural sounds. Sounds of speech (top) and rain (bottom) are shown. ***b***, Modulation spectra showing the distributions of the AM components of the sounds in ***a***. The modulation spectrum was calculated as the RMS of the filtered envelope with a logarithmically spaced band-pass filter bank. Each modulation spectrum was normalized by its maximum value. The lower and upper peaks in the modulation spectrum of speech (top) probably contain information about the speech content and the speaker, respectively. The modulation spectrum of the rain sound (bottom) appeared different from that of speech.

Natural sounds such as speech and environmental sounds exhibit rich patterns of amplitude modulation (AM) (Fig. 1*a*) (Varnet et al., 2017). For example, humans can recognize speech content and identify daily sounds based on their AM patterns even if the fine temporal structure is substantially degraded (Dudley, 1939; Shannon et al., 1995; Gygi et al., 2004). The AM rate (i.e., the number of AM cycles per second) is one of the most important physical dimensions in auditory perception (Fig. 1*b*) (Houtgast and Steeneken, 1985). Psychophysical studies suggest that the sensitivity of the auditory system to AM can be accounted for by filtering mechanisms in the AM rate domain (Viemeister, 1979; Bacon and Grantham, 1989; Houtgast, 1989; Dau et al., 1997).

The auditory system converts the physical properties of a sound stimulus to neural activities and processes them through a cascade of brain regions (see Fig. 30–12 in Kandel et al., 2000). Neurophysiological studies have found a number of neurons and neural populations that tune to the AM rate (Giraud et al., 2000; Joris et al., 2004; Liégeois-Chauvel et al., 2004; Sharpee et al., 2011). Some neurons fire synchronously with the stimulus AM waveform and the degree of synchrony depends on the AM rate (temporal coding of AM rate), whereas others encode the AM rate with their firing rates (rate coding of AM rate). Interestingly, the characteristics of AM tuning in temporal and rate coding transform systematically along the processing stages from the periphery to the cortex (Joris et al., 2004; Sharpee et al., 2011): the AM rate with which neurons synchronize gradually decreases and the number of neurons that perform rate coding gradually increases (a phenomenon known as time-to-rate conversion).

Neurophysiological and theoretical studies have revealed how the auditory system works by exploring the neural mechanisms of the transformation of AM tuning (Hewitt and Meddis, 1994; Depireux et al., 2001; Zhang and Kelly, 2003; Guérin et al., 2006; Dicke et al., 2007; Mahajan et al., 2014). However, the functional significance of the transformation remains unknown. In other words, we still face the question of why the system has to be organized in that way.

### Functional model of sensory systems

A computational approach with machine learning techniques is effective for explaining functional significance in sensory systems (Olshausen and Field, 1996; Lewicki, 2002; Terashima and Okada, 2012; Młynarski and McDermott, 2017). The architectures and parameters of the models are trained to process natural stimuli for behaviorally relevant objectives with few assumptions regarding anatomical or physiological properties. Therefore, the trained model is expected to provide an effective representation of natural stimuli and if the representation is similar to that observed in a real sensory system, it is highly likely that the sensory system is also adapted to effectively processing sensory information for survival. In particular, a deep neural network (DNN) is one of the most successful models for both automatic data processing (Hinton et al., 2012; Krizhevsky et al., 2012; Schmidhuber, 2015) and explaining a neural representation of sensory information (Khaligh-Razavi and Kriegeskorte, 2014; Yamins et al., 2014; Horikawa and Kamitani, 2017; Zhuang et al., 2017; Cueva and Wei, 2018; Kell et al., 2018). A DNN consists of a cascade of layers with multiple units and a unit in a layer integrates the activations in a lower layer, which makes the model suitable for explaining the functions of cascaded brain regions.

In the present study, we optimized a DNN for natural sound recognition. To make a direct comparison of AM sensitivity in the DNN and that in the auditory system reported in a number of neurophysiological studies, we characterized the AM sensitivity of the DNN using standard neurophysiological methods by treating the DNN as if it were a biological brain. We showed the qualitative and quantitative similarities of the DNN to the auditory system.

## Materials and Methods

### 

#### 

##### Task.

The task of the DNN was sound recognition. Specifically, the task was to estimate the sound category at the last time frame of a sound of a certain duration (0.19 s for natural sounds and 0.26 s for speech). Classification accuracy is defined as the average correct classification rate for each category, namely the number of time frames correctly estimated as a particular category divided by the total number of time frames in the category.

##### Datasets.

Two datasets were used to train the DNNs. The first consisted of nonhuman natural sounds and is a subset of ESC-50 (Piczak, 2015). The original dataset contains 50 sound categories with 40 sounds for each category. From the original dataset, we used 18 categories of sounds not produced by human activity. Each entry in the original dataset contains a sound waveform with a length of <5 s and the sound category. In this study, we excluded silent intervals, resulting in a total length of 53.9 min. The original dataset is divided into five folds for cross-validation. We used fold #5 for validation and the other folds for training. The sound format was converted to 44.1 kHz 16-bit linear PCM.

The second dataset consisted of speech sounds (Garofolo et al., 1993). Each entry in the dataset contains the sound waveform of a single spoken sentence, categories of vocal elements, and the time intervals of each element. There were originally 61 categories. We merged some categories in accordance with previous studies (Lee and Hon, 1989; Lopes and Perdigao, 2011), resulting in 39 categories. The average and total durations of the sound were 3.1 s and 3.3 h, respectively. The data were originally divided into a validation set and a training set. In this study, we followed the original division. The validation and training sets contain the speech of 24 and 462 speakers, respectively. The speakers and sentences in the two divisions do not overlap. The sound format was 16 kHz 16-bit linear PCM.

##### Network architecture.

**Figure 2.**
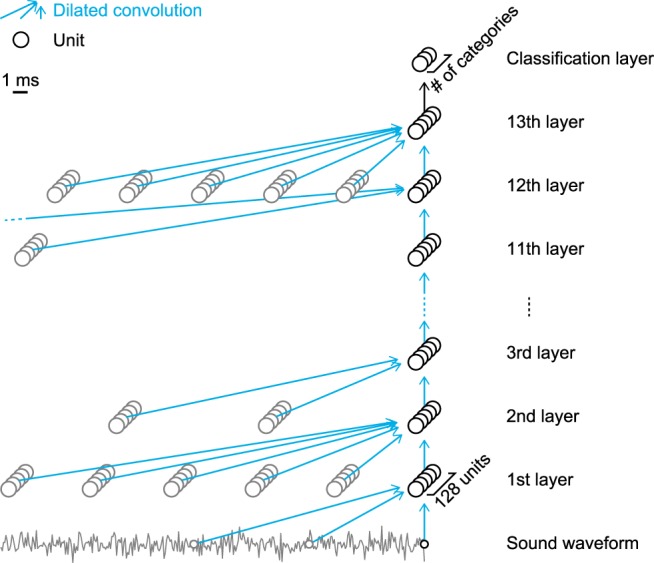
DNN architecture. Our DNN consists of a stack of one-dimensional dilated convolutional layers. The figure shows the architecture of the DNN for natural sounds. Each layer contains 128 units and performs dilated convolution followed by a nonlinear activation function. The first layer takes a raw sound waveform as an input and the highest layer is connected to the classification layer, which was excluded from the analysis. The output is the category label assigned to the classification unit with maximum activation. We tested multiple architectures with random filter and dilation lengths in each convolutional layer and selected the DNN that achieved the best classification accuracy on the novel dataset. The filter and dilation lengths in all the layers are shown in Table 1. The numbers of layers and units in each layer were chosen in the pilot experiment.

Our DNN consisted of a stack of dilated convolutional layers (van den Oord et al., 2016) (Fig. 2), in which convolutional filters were evenly dilated in time. Convolution was conducted along the time axis. Each layer performed a dilated convolution on the activations of the previous layer and applied an activation function. The activation function was an exponential linear unit (Clevert et al., 2016). The first layer took samples of raw waveforms directly as an input. Each layer contained multiple units. All of the layers contained the same number of units for simplicity. The units in the highest layer were connected to the classification layer without convolution. The number of units in the classification layer was the same as the number of categories. The classification layer was omitted from the physiological analysis.

We used DNNs with 13 layers, each containing 128 units, for nonhuman sound and DNNs with 12 layers, each containing 64 units, for speech. The number of layers and the number of units in each layer were determined based on a pilot study and fixed throughout the study. In the pilot study, DNNs with various numbers of layers and units were trained using a random portion of the training set. The filter length was 2 and the dilation length was 2 to the power of the layer number (van den Oord et al., 2016). The number of layers and the number of units in each layer that gave the best classification accuracy on the other portion of the training set were used in the following study.

We tested multiple architectures with random filter sizes and dilation lengths in each convolutional layer and selected the DNN that achieved the best classification accuracy on the novel dataset (Table 1). The filter size and dilation length were randomly chosen for each layer with certain constraints, namely that the filter size did not exceed 8 and the total input length for the whole DNN, which is equal to the length of the input time window of the topmost layer, did not exceed 8192 (∼0.19 s) for nonhuman sound and 4096 (∼0.26 s) for speech. The number of layers and the number of units in each layer were fixed as mentioned in the previous paragraph.

**Table 1.** DNN architecture

Layer no.	No. of channels	Dilation width	Filter width
13	128	109	6
12	128	594	3
11	128	167	8
10	128	180	6
9	128	564	3
8	128	204	6
7	128	70	5
6	128	68	8
5	128	4	8
4	128	6	4
3	128	226	3
2	128	123	6
1	128	174	3

##### Optimization.

The DNNs were trained on the training set and the classification accuracy was calculated for the validation set. The initial filter weights were randomly sampled and the biases were set at 0 in accordance with a previous study (He et al., 2015). The filter weights and biases were updated using the Eve algorithm (Koushik and Hayashi, 2016) with softmax cross entropy as the cost function. The number of iterations for a parameter update was determined as the value that gave the best classification accuracy on a random portion of the training set trained on the rest of the training set.

##### Experimental design: physiological analysis of a DNN.

For a physiological analysis of a DNN, a sound stimulus was fed to the DNN and the values of each unit were recorded. The root mean square (RMS) of the input sound was adjusted approximately to the RMS of the training set. Before the analysis, 1 was added to the values of all the units because the minimum possible value of the activation function is −1 (Clevert et al., 2016).

The stimulus was 8 s of sinusoidally amplitude-modulated white noise. In physiological studies, tuning to an AM rate is measured using sinusoidally amplitude-modulated tones with carriers at the neurons' best acoustic frequency (AF, frequency of a sound waveform itself but not its amplitude envelope), sinusoidally amplitude-modulated white noises, or click trains. We did not use tones as carriers because many units showed multiple troughs in the AF tuning curves or nonmonotonic responses to the input amplitude, making it difficult to define the best AFs.

The synchrony to the stimulus and the average activity was calculated from the activations of each unit. The synchrony to the stimulus was quantified in terms of vector strength (Goldberg and Brown, 1969). When dealing with spike timing data recorded in neurons, each spike is represented as a unit vector with its angle corresponding to the modulator phase at that time and the vector strength is defined as the average length of these unit vectors. Equivalent operations were applied to the continuous output of the DNN unit to derive the vector strength (Eq. 1). The vector strength had a value between 0, indicating no synchrony, and 1, indicating perfect synchrony, as follows:

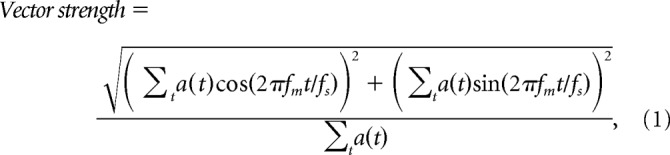
 where *t* is an index of the time frame, *a*(*t*) is the unit activation, *f*_s_ is the sampling rate, and *f*_m_ is the stimulus AM rate. The average activity was defined as the temporal average of the values, which could be considered as the DNN version of an average spike rate. The synchrony and the average activity were averaged for 16 instances of the carrier white noise to reduce the effect of stimulus variability. A temporal modulation transfer function (tMTF) and a rate MTF (rMTF) were defined as the synchrony and average activity as functions of the AM rate, respectively. In physiology, an MTF is usually defined only at the AM rates at which the unit shows a statistically significant synchrony or spike rate. Because a statistical test on the results of a deterministic model such as our DNN makes no sense, we considered the synchrony or average activities below a certain threshold as “nonsignificant” and excluded them from the following analysis. The threshold was arbitrarily set at 0.01 for the synchrony and at 0.01 above the average activity in response to unmodulated white noise for the average activity.

An MTF was classified as one of the following four types: low-pass, high-pass, band-pass, or flat. A low-pass type MTF was defined as one that had no values smaller than 80% of its maximum for AM rates smaller than the peak rate. A high-pass type MTF was defined as one that had no values smaller than 80% of its maximum for rates larger than the peak rate. A flat MTF was defined as one that had no values smaller than 80% of its maximum or one with a peak to peak range of <0.1. The band-pass MTF was defined as being other than the above.

The best modulation frequencies (BMFs) were calculated from the band-pass type MTFs and the upper cutoff frequencies (UCFs) were calculated from the low-pass and band-pass type MTFs. The BMFs of low-pass, high-pass, or flat MTFs and the UCFs of high-pass or flat MTFs were considered to be impossible to define. The BMF was defined as the modulation rate at the peak of the MTF. If there were multiple peaks with the same height, the geometric mean of the rates was taken. The UCF was calculated in two different ways: one for qualitative visualization as shown in Figure 7*a* and the other for quantitative comparisons with specific physiological data for neurons found in the literature. The UCF for visualization was defined as the rate at which the MTF crosses 80% of its maximum value. If an MTF had multiple such rates, the geometric mean of the rates was used. The threshold of the UCF used for a quantitative comparison with the auditory system varied according to the reference physiology study. The thresholds were 50% (Eggermont, 1998; Zhang and Kelly, 2006), 80% (Rhode and Greenberg, 1994; Kuwada and Batra, 1999), and 70% (−3 dB) (Joris and Yin, 1992, 1998; Joris and Smith, 1998) of the maximum, 90%:10% interior division of its minimum and maximum (Krishna and Semple, 2000), an absolute value of 0.1 (Rhode and Greenberg, 1994; Zhao and Liang, 1995), and the highest rate that gives significant responses (Batra et al., 1989; Kuwada and Batra, 1999; Krishna and Semple, 2000; Lu and Wang, 2000; Lu et al., 2001; Liang et al., 2002; Batra, 2006; Bartlett and Wang, 2007; Scott et al., 2011). If there was no rate at which the MTF crossed the threshold, then the UCF was considered to be impossible to define.

The stimuli we used for calculating AF tuning were tones with various AFs and amplitudes. The activation of each unit was temporally averaged to obtain the response to a particular stimulus. The tuning curve was defined for each AF as the smallest amplitude inducing a response larger than a certain threshold. In physiological studies, thresholds are usually determined arbitrarily. Figure 15 shows tuning curves with thresholds of 0.001, 0.01, and 0.1.

##### Comparison with auditory system.

We extracted the BMF and UCF distributions reported in previous physiological studies by digitizing the figures. If multiple figures were available, then we chose the clearest figure or that with the most neurons. The extracted values were used for a qualitative visualization in Figure 7*c* and a quantitative comparison with the DNNs. For the visualization, we averaged the distributions of all the subregions and all the neuron types in each region in each study. Then, the distributions in all the papers were averaged for each region. The resulting distributions were smoothed with a Gaussian filter with a width of 0.136 on a logarithmic scale of base 10. For quantitative comparison with a DNN, we calculated the similarity of each extracted distribution to the distribution of each layer in the DNN. As the measure of similarity, we used the Kolmogorov–Smirnov statistic subtracted from 1 because it is nonparametric and does not depend greatly on the bin widths of the histogram. For each BMF and UCF for each rate and temporal coding, we averaged the similarities in the same regions in a single study and then averaged the similarities in the same region in different studies. Averaging the four pairwise similarities (tBMF, tUCF, rBMF, and rUCF), we derived the final layer–region pairwise similarity matrix. Because no distribution of tBMF has been reported in auditory nerves (ANs); no distribution of rBMF has been reported in ANs, cochlear nucleus (CN), or superior olivary complex (SOC), and no distribution of rUCF has been reported in AN or CN, their similarities were set at 1 if there was no unit with a definable BMF or UCF and set at 0 otherwise. For regions other than these, the similarity was set at 0 if there was no unit with a definable BMF or UCF.

##### Evaluation of a pairwise similarity matrix.

The similarity of the entire cascade and that of each layer were calculated from a pairwise similarity matrix. We wanted to evaluate the pairwise similarity matrix in a way in which high scores are obtained by a DNN with its lower, middle, and upper layers being similar to the peripheral, middle, and central brain regions, respectively. To realize this evaluation concept, we defined the cascade similarity as the weighted mean of the pairwise similarity matrix. The weight at the cell (*i*, *j*) was proportional to the following:

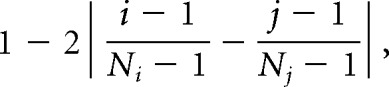
 where *N*_i_ and *N*_j_ are the number of brain regions (7) and the number of DNN layers, respectively. The weight was scaled so that the squared mean of the weight matrix was 1. The weight was maximal on the diagonal line and minimal in the top left and bottom right corners. The layerwise similarity was defined as the mean obtained in each layer.

##### Control experiments.

In the first control experiment, the category labels of the sounds in the training set were randomly shuffled. The validation set was not modified. A parameter update was conducted for the same number of iterations as the original nonrandom condition. In the second control experiment, the waveform in each sound in the training set was randomly permuted, resulting in a noise-like input waveform maintaining only the marginal distribution of the amplitudes. The third control experiment was a waveform following task that involved copying the amplitude value of the last time frame of the input sound segment. To make the result directly comparable to those of the classification tasks, the target amplitude was quantized and we used a softmax cross entropy cost function (van den Oord et al., 2016). The waveform was nonlinearly transformed with a μ-law companding transformation before quantization (van den Oord et al., 2016). The number of bins was equal to the number of sound categories in the original classification task.

##### Sharpness of a tMTF.

The Q factors of tMTFs were calculated as in a previous physiological study (Rodríguez et al., 2010) as follows:

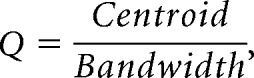


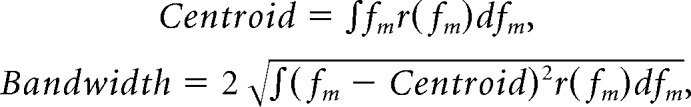

where *f*_m_ and *r*(*f*_m_) denote the AM rate and tMTF, respectively. Integration was calculated with the trapezoidal rule. Q factors were calculated only in units with a band-pass tMTF.

##### Statistical analysis.

Spearman's rank correlation coefficients were calculated with the recognition performance and the cascade similarity. The sample size was 100 when comparing them in relation to optimization progress and 39 when comparing them across different model architectures.

##### Data availability.

The datasets used for the training and validation of the model are available from the cited studies (Garofolo et al., 1993; Piczak, 2015). The model architecture is available in Table 1. Source codes for training, evaluation, and physiology of DNNs are available at https://github.com/cycentum/cascaded-am-tuning-for-sound-recognition. Trained models and recorded activities are available at https://doi.org/10.6084/m9.figshare.7914611.

## Results

### Functional model of the auditory system

**Figure 3.**
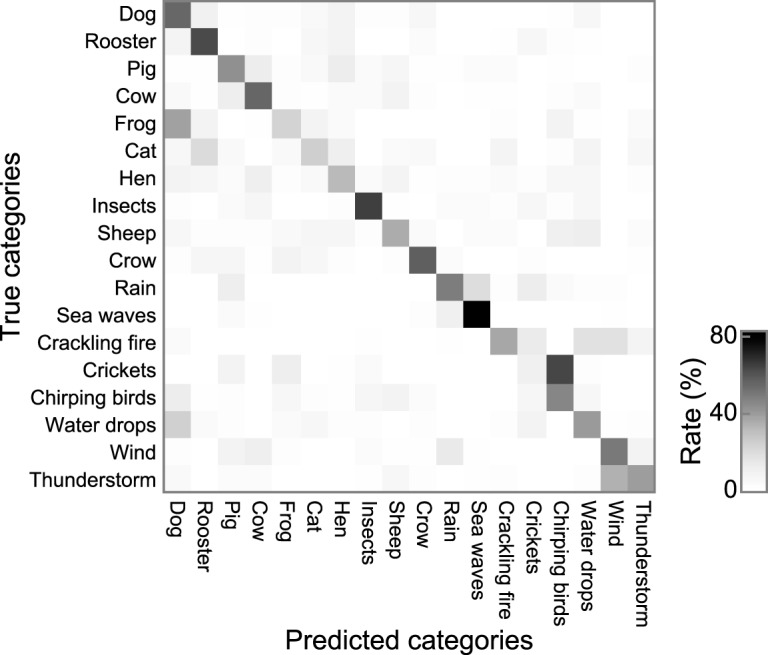
Confusion matrices of classification of validation data. There are 18 categories. The labels of the true categories are shown in the ordinates and those of the predicted categories are shown in the abscissas. The value in each cell is calculated as the time frame fractions classified as a particular category among the total number of time frames with the true category. Cells with a high classification rate are in the diagonal of the matrices, indicating high classification accuracy. The classification accuracy was defined as the mean values in the diagonal of the matrix.

**Figure 4.**
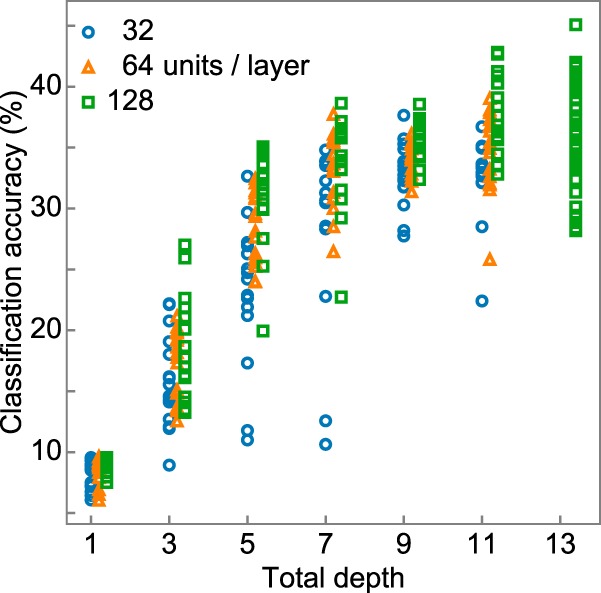
Importance of the deep cascade. Classification accuracy of DNNs with various numbers of layers with random filter and dilation lengths. Models with 1, 3, 5, 7, 9, 11, and 13 layers were tested. We tested 32 (blue circles), 64 (orange triangles), and 128 (green squares) channels. DNNs with 13 layers and 32 or 64 channels were not tested because they were excluded by the pilot study. The deeper the DNN, the higher the classification accuracy appeared to be. The result indicates the importance of the deep cascade.

The DNN was trained to classify raw sound data (i.e., amplitude waveforms) of nonhuman natural sounds consisting of animal vocalizations and environmental sounds. Therefore, the model covers the entire range of auditory processes from the stage in the ear to the final recognition (Fig. 2). This makes our model suitable for explaining the entire cascade of the auditory system with as few assumptions as possible. This is in contrast to typical auditory studies, which assume frequency-decomposed inputs such as spectrograms. The classification accuracy of the optimized DNN was 45.1% (Fig. 3). We confirmed that a deep cascade is necessary to achieve high classification accuracy (Fig. 4). Although the classification accuracy was not as good as that reported in other studies (Aytar et al., 2016), this difference in performance is reasonable when we consider that the previous studies used much longer sound segments than ours.

### Emerging tuning to AM rate

**Figure 5.**
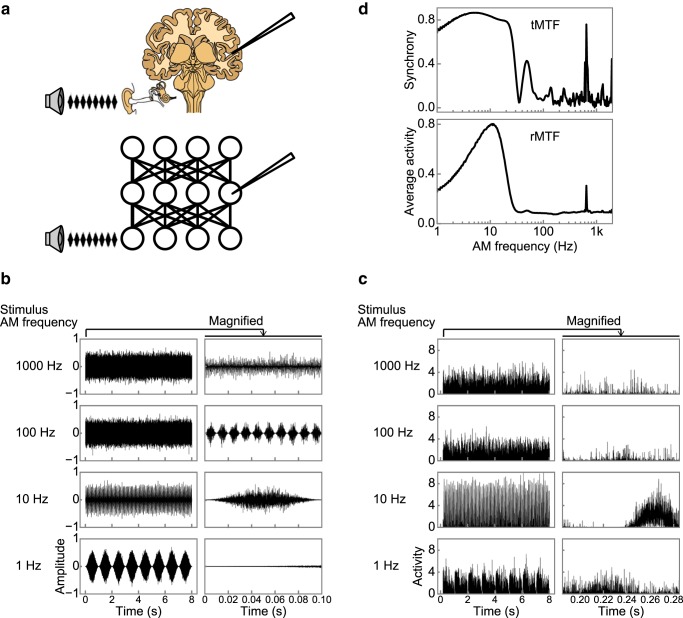
Single-unit recording in the DNN. ***a***, Illustrations of single-unit recording in a brain (top) and in a DNN (bottom). In physiological experiments, neural activities are recorded while presenting an AM sound stimulus to the animal. We simulated the method and recorded the unit activities of the DNN while feeding it an AM sound stimulus. ***b***, Examples of AM stimuli with 1, 10, 100, and 1000 Hz AM rates. The carrier was white noise. Temporally magnified plots are shown on the right. ***c***, Examples of responses to the AM stimuli in ***b*** in a single unit. A unit in the eighth layer is chosen as an example. ***d***, An example of tMTF (top) and rMTF (bottom) in the same unit as in ***c***. A tMTF and an rMTF are defined as synchrony with the stimulus AM rate and the average activity as functions of AM rate, respectively. The unit exhibited the low-pass type tMTF and the band-pass type rMTF.

To enable a direct comparison of our DNN and the auditory system, we simulated the experimental approaches of typical neurophysiological studies. Specifically, we conducted “single-unit recording” for each unit in the DNN while presenting a sinusoidally amplitude-modulated sound stimulus (Fig. 5*a*,*b*). A single unit responded differently to stimuli with different AM rates (Fig. 5*c* shows examples). We characterized the tuning of the units' activities to AM rate in terms of temporal and rate coding with tMTF and rMTF (Joris et al., 2004), namely the synchrony and the average activity as functions of the AM rate, respectively (Fig. 5*d*).

**Figure 6.**
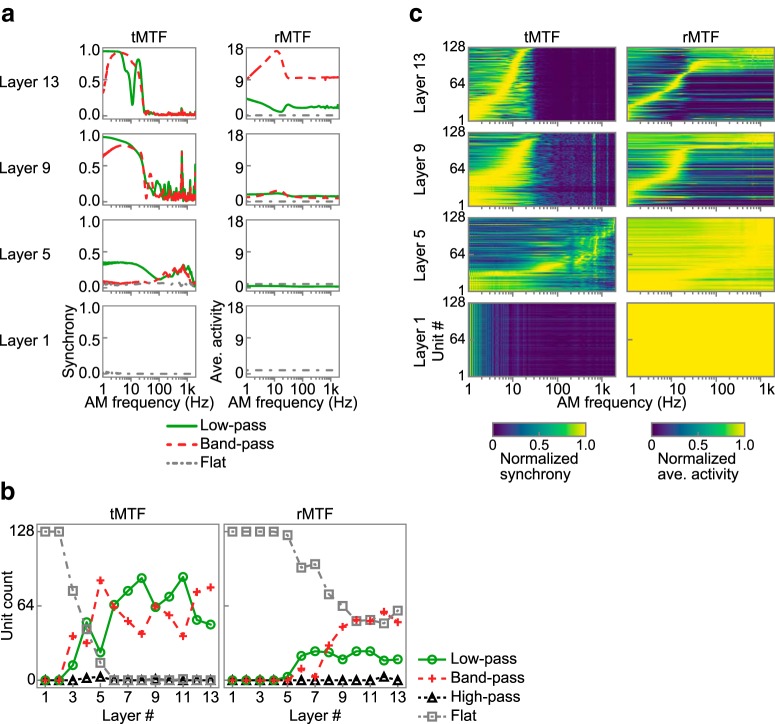
Emergent AM tunings in the DNN. ***a***, Examples of tMTFs (left), and rMTFs (right) in the first, fifth, ninth, and 13^th^ layers. The layers are sorted vertically from bottom to top. One example of a low-pass (solid green line), a band-pass (dashed red line), and a flat (dash-dotted gray line) MTF is shown for each layer. ***b***, Number of units with the low-pass (solid green lines with circles), band-pass (dashed red lines with crosses), high-pass (dotted black lines with triangles), and flat (dash-dotted gray lines with squares) type tMTFs (left) and rMTFs (right). ***c***, Heat maps of all tMTFs (left) and rMTFs (right) in the first, fifth, ninth, and 13^th^ layers. The MTFs are normalized by their peak values for better visualization. The units are sorted vertically by their peak AM rates. In some layers, distinct peaks and notches appeared commonly across different units at particular AM rates (observed as vertical lines in tMTFs). We have no clear explanation for these features, but they are probably due to artifacts of discrete convolutional operation.

Figure 6*a* shows MTFs of representative units in the first, fifth, ninth, and 13th layers. As in typical physiological experiments, we classified the MTFs into low-pass, band-pass, high-pass, or flat types according to certain criteria. Most units exhibited low-pass, band-pass, or flat MTFs (Fig. 6*b*). All of the MTFs in the first layer were flat, indicating that the first layer did not tune to AM rates. In the fifth layer, units with low-pass or band-pass tMTFs appeared and a very small number of units with low-pass rMTFs were observed. In the ninth and higher layers, the tMTF magnitude generally increased and the number of units with low-pass or band-pass rMTFs also increased. Heat maps of all tMTFs normalized by their peaks reveal a downward shift of the distribution of the preferred AM rates from the fifth layer to the highest layer and distinct tuning in the rMTFs appearing in the ninth layer and above (Fig. 6*c*).

### Comparison with the auditory system

**Figure 7.**
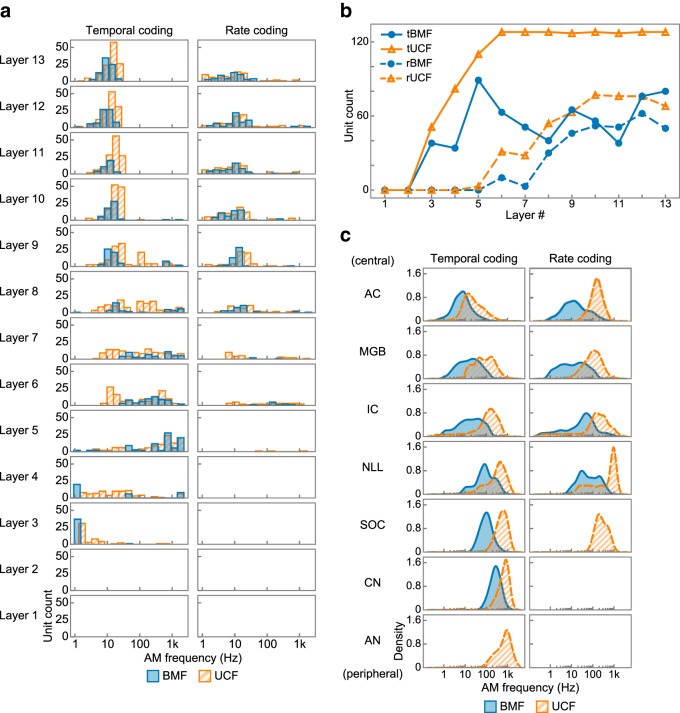
Similar distributions of MTF shapes in the DNN and those in the auditory system. ***a***, Histograms of BMF (filled blue bars) and UCF (hatched orange bars) of temporal (left) and rate (right) coding in each layer. The layers are sorted vertically from bottom to top. ***b***, Number of units with a definable BMF (filled blue circles) and UCF (open orange triangles) of temporal (solid lines) and rate (dashed lines) coding. ***c***, Distributions of BMF (filled blue areas) and UCF (hatched orange areas) of the temporal (left) and rate (right) coding in each region in the auditory system. Regions are sorted vertically from the peripheral region (bottom) to the central region (top). No distribution is drawn where none is reported.

As in typical neurophysiological studies, the MTF of a unit was characterized by its BMF, the AM rate at which a neuron exhibits the largest synchrony or average activity, and its UCF, the AM rate at which the synchrony or average activity starts to decrease. The BMF and UCF of temporal and rate coding are denoted tBMF/tUCF and rBMF/rUCF, respectively. In the first and second layers, no BMFs or UCFs were definable because all MTFs were flat (Fig. 7*a*,*b*). In the third and fourth layers, some units exhibited definable tBMFs and tUCFs, but no rBMFs or rUCFs were definable. In the fifth layer, the tBMFs and tUCFs appeared to be high and a small number of units exhibited definable rBMFs and rUCFs. When ascending the layer cascade from the fifth layer, the mode tBMF/tUCF decreased and the number of units with definable rBMFs/rUCFs increased. In summary, the distributions of the tBMFs and tUCFs shifted toward lower AM rates when ascending from the middle to the high layers (Fig. 7*a*, left) and the units that code AM rate by their average activities appear only in the higher layers (Fig. 7*a*, right, 7*b*).

The transformation of the BMF/UCF distributions reminds us of the well known characteristics of the auditory pathway, namely the decrease in synchronizing AM rate and the time-to-rate conversion of AM coding (Joris et al., 2004; Sharpee et al., 2011). Figure 7*c* depicts the distributions of BMFs and UCFs in the auditory system by combining previously reported distributions for each of the seven brain regions: ANs (Joris and Yin, 1992; Rhode and Greenberg, 1994), the CN (Frisina et al., 1990; Rhode and Greenberg, 1994; Zhao and Liang, 1995; Joris and Smith, 1998; Joris and Yin, 1998), the SOC (Joris and Yin, 1998; Kuwada and Batra, 1999), the nuclei of the lateral lemniscus (NLL) (Huffman et al., 1998; Batra, 2006; Zhang and Kelly, 2006), the inferior colliculus (IC) (Müller-Preuss, 1986; Langner and Schreiner, 1988; Batra et al., 1989; Condon et al., 1996; Krishna and Semple, 2000), the medial geniculate body (MGB) (Preuss and Müller-Preuss, 1990; Lu et al., 2001; Bartlett and Wang, 2007), and the auditory cortex (AC) (Müller-Preuss, 1986; Schreiner and Urbas, 1988; Bieser and Müller-Preuss, 1996; Schulze and Langner, 1997; Eggermont, 1998; Lu and Wang, 2000; Liang et al., 2002; Scott et al., 2011; Yin et al., 2011). In the peripheral regions, the tBMFs and tUCFs cluster around high AM rates and, as they ascend toward the central region, the mode rates decrease. rBMFs are only reported in the NLL or above and rUCFs are reported in the SOC or above. This meta-analysis suggests that the distributions of the BMF and UCF in the DNN and those in the auditory system are qualitatively similar.

**Figure 8.**
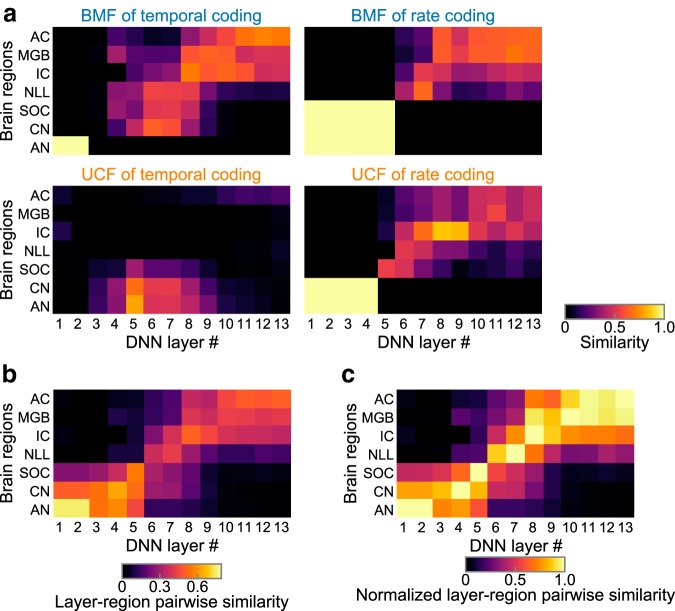
Similarity to the auditory system throughout the entire cascade revealed by the layer–region pairwise similarity. ***a***, Layer–region pairwise similarities of BMF (top) and UCF (bottom) of temporal (left) and rate (right) coding. The four pairwise similarities were averaged to yield the final layer–region pairwise similarity shown in ***b***. In all of them, the lower, middle, and upper layers appeared to be similar to the peripheral, middle, and central brain regions, respectively, although the similarities are not as smooth or clear as their average. ***b***, Layer–region pairwise similarity of the AM representation in the DNN layers (horizontal axis) and that in the regions in the auditory system (vertical axis). ***c***, Layer–region pairwise similarity normalized by the maximum value of each brain region.

Next, we compared those distributions quantitatively. For each tBMF, tUCF, rBMF, and rUCF, we calculated the similarity between the distribution in each layer of the DNN and the distribution in each region in the auditory system (Fig. 8*a*) and averaged them to yield the layer–region pairwise similarity (Fig. 8*b*). Pairs consisting of a DNN layer and a brain region with a large similarity appeared in the diagonal direction, indicating that lower, middle, and higher DNN layers are similar to the peripheral, middle, and central brain regions, respectively. This similarity of the entire cascade is more clearly observed if we normalize the pairwise similarity by the maximum value in each brain region (Fig. 8*c*).

### Relationship with optimization

**Figure 9.**
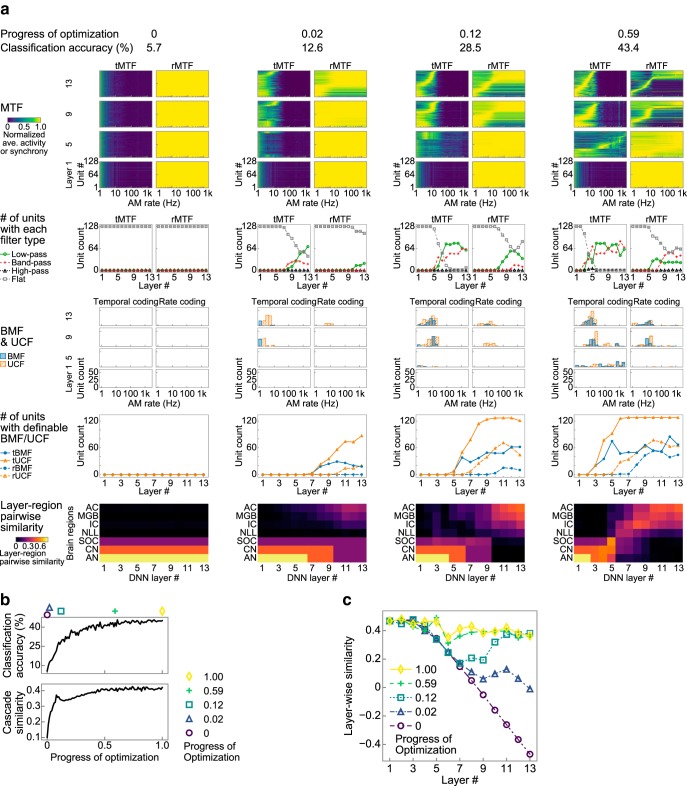
Development of AM representation in the DNN during optimization. ***a***, From top to bottom: heat maps of all tMTFs (left) and rMTFs (right) in the first, fifth, ninth, and 13^th^ layers (as in Fig. 6*c*); the number of units with low-pass, band-pass, high-pass, and flat MTFs (as in Fig. 6*b*); histograms of BMFs and UCFs of temporal (left) and rate (right) coding (as in Fig. 7*a*); number of units with definable tBMF, tUCF, rBMF, and rUCF (as in Fig. 7*b*); and layer–region pairwise similarity (as in Fig. 8*b*). The progress of the optimization and the classification accuracy is shown at the top of each column. Auditory-system-like AM tuning gradually emerged as optimization progressed. ***b***, Classification accuracy (top) and cascade similarity (bottom) as functions of the progress of optimization. The progress of optimization, shown on the horizontal axis, is linearly scaled so that the value is 1 at the end of the optimization. Colored markers indicate the points at which the layerwise similarities were calculated in ***c***. ***c***, Layerwise similarity at four intermediate snapshot instances during optimization. Colors, markers, and lines indicate the progress of optimization as indicated by the legend and in ***b***.

The similarity of the entire cascade could be due to the convolutional architecture inherent to the DNN (Saxe et al., 2011) or to optimization for sound recognition. To test these possibilities, we measured the MTFs in the DNN before and during optimization. Before optimization, no unit exhibited clear selectivity in regard to AM rate and there was no transformation of the MTFs across layers (Fig. 9*a*, left).

**Figure 10.**
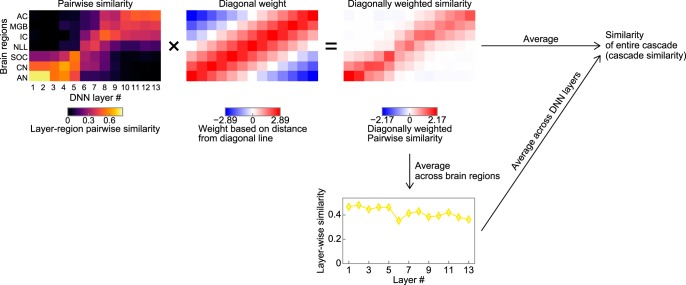
Evaluation of the similarity of the entire cascade. The cascade similarity was defined as the weighted mean of the pairwise similarity matrix. The weight was designed to be larger near the diagonal line and smaller in the top left and bottom right corners. The layerwise similarity was defined as the mean calculated across brain regions within each layer.

As the optimization progressed, the classification accuracy increased as expected (Fig. 9*b*, top). Auditory-system-like AM tuning gradually emerged in parallel (Fig. 9*a*). We evaluated the similarity over all the cascades by measuring the degree of diagonality of the pairwise similarity matrix (Fig. 10) and we refer to this as the cascade similarity. A large cascade similarity value indicates that, in the pairwise similarity matrix, cells around a diagonal line exhibit a large similarity and cells around the top left and bottom right corners exhibit a small similarity. The cascade similarity increased as the optimization progressed (Fig. 9*b*, bottom) and correlated very well with the classification accuracy (Spearman's rank correlation coefficient = 0.84, *p* = 8.57 × 10^−28^, *n* = 100). The results indicate that the AM representation in the DNN emerged during the optimization.

**Figure 11.**
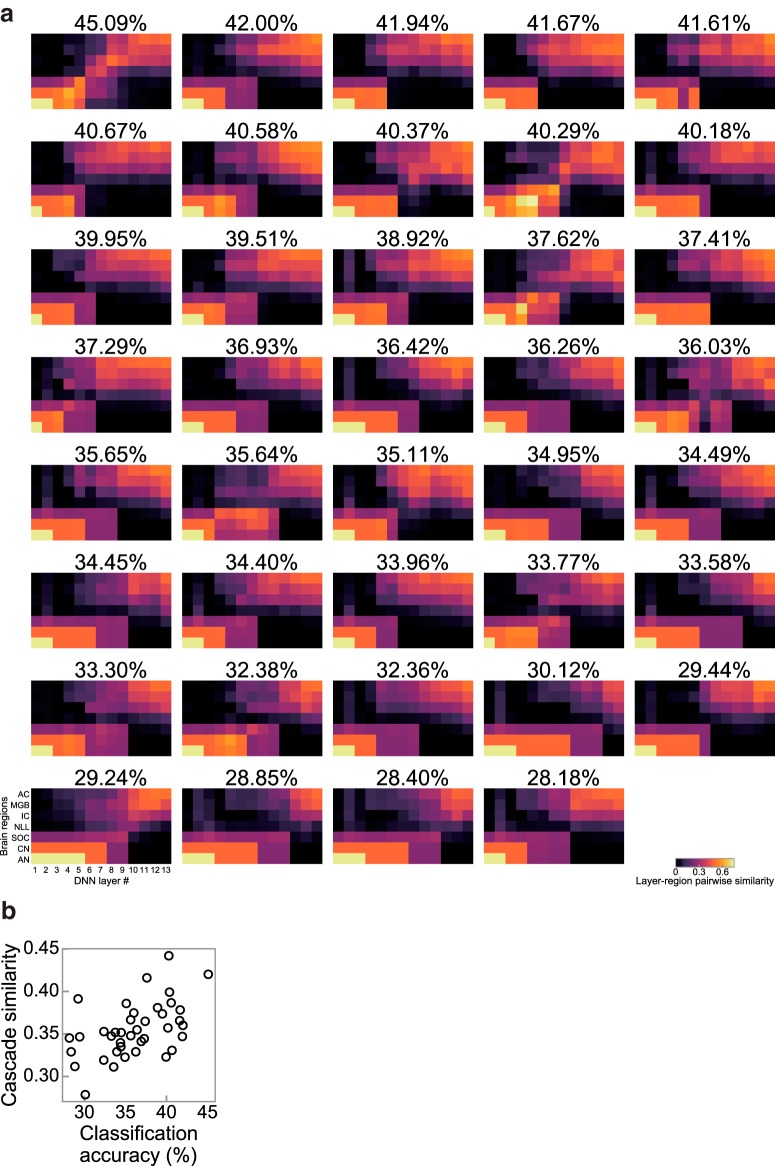
Cascade similarity of DNNs with various architectures correlated with their classification accuracy. ***a***, Heat maps showing the layer–region pairwise similarity sorted in terms of classification accuracy, which is shown at the top of each panel. The top left panel is identical to Figure 8*b*. Pairwise similarities along a diagonal line appeared larger in DNNs with high classification performance. ***b***, Cascade similarities of DNNs with various architectures plotted against their classification accuracies. A single circle represents a single architecture.

Because the classification accuracy of a DNN generally depends on its architecture (Bergstra and Bengio, 2012; Bergstra et al., 2013), so could its cascade similarity (Yamins et al., 2014; Kell et al., 2018). We trained DNNs with various architectures and examined them using the same physiological analysis. The classification accuracy of these DNNs varied between 28.2% and 45.1%. The patterns of the layer–region pairwise similarity also varied among the architectures (Fig. 11*a*) and the cascade similarity correlated with the classification accuracy (Fig. 11*b*; ρ = 0.51, *p* = 8.08 × 10^−4^, *n* = 39). The results indicate that AM representation in better-recognizing DNNs have a greater similarity to that in the auditory system. The similarity to the auditory system correlated with the classification accuracy across both different model parameters and different architectures, which suggests that the auditory AM representation is strongly related to task optimization but not to the convolutional operation alone.

### Different factors for different regions

The development of the layer–region pairwise similarity during optimization indicates that an auditory-system-like AM representation is initially exhibited only in the lower layers and that, as the optimization progresses, it first emerges in the upper layers and then in the middle layers (Fig. 9*a*). This pattern was more clearly seen when we calculated the similarity to the auditory system in each layer, which we refer to as layerwise similarity (Figs. 9*c*, 10). The results imply that multiple factors can underlie these across-layer differences in the developmental patterns. To isolate the possible factors in each region, we conducted three control experiments expecting to see different degrees of similarity emerge in different layers depending on the control conditions.

**Figure 12.**
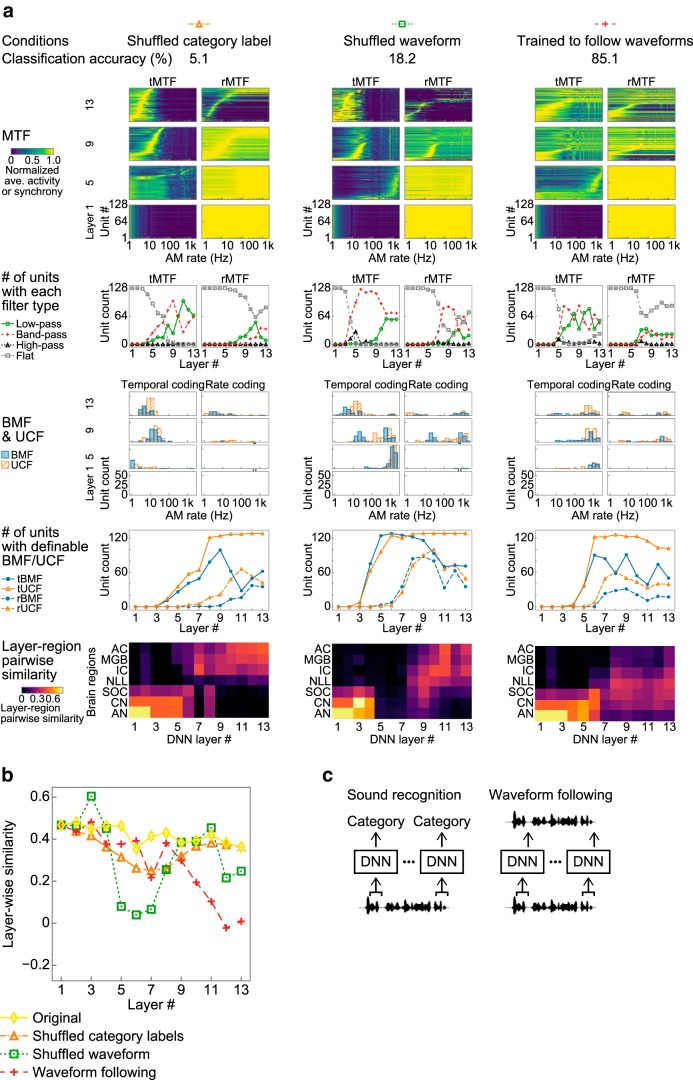
AM representation in DNN with control conditions. ***a***, AM representation in a DNN trained on shuffled category labels (left column), on shuffled waveform (middle column), and optimized for the waveform following task (right column). Colored symbols and lines by the panel titles indicate the types of control condition as in ***b***. Other conventions are the same as in Figure 9*a*. ***b***, Layerwise similarity in the control experiments. The similarities under the original condition (yellow diamonds and solid line) are also shown. ***c***, Schematic illustration of recognition and waveform following tasks. In both tasks, the DNN operated on a short sound segment. The sound recognition task was to estimate the category of the input sound. The waveform following task was to copy the amplitude value of the last time frame of the input segment.

The first two control experiments tested the effect of the data structure. It has been shown that a DNN is capable of learning the input–output correspondence even when trained on data with random category labels or data without natural statistics (Zhang et al., 2017). Under the first condition, the input–output correspondence was destroyed by shuffling the category labels. Under the second condition, the structure of the input waveform was destroyed by shuffling the amplitude values of each waveform. Under this condition, the DNN was able to classify the novel sounds with some accuracy probably because the shuffled waveform retained its overall amplitude distribution, although both the frequency and temporal statistics were completely destroyed. The DNNs trained under these two conditions exhibited an auditory-system-like AM representation in the lower and upper layers, but not in the middle layers (Fig. 12*a*,*b*, orange triangles and green squares). When the DNN was trained on shuffled labels, very few units in the middle layers appeared to exhibit AM tuning (Fig. 12*a*, left column). When the DNN was trained on shuffled waveforms, units in the middle layers appeared to exhibit some AM rate tuning, but they synchronized with a much higher AM rate than neurons in the auditory system, resulting in the upper layers being similar to the middle brain regions (Fig. 12*a*, middle column). The results indicate that a midlevel AM representation requires a natural data structure, although low-level and high-level representations could emerge even by optimization with unnatural data.

The third control experiment examined the effect of the optimization objective. A DNN can be optimized for behaviorally irrelevant objectives such as a waveform following task (Fig. 12*c*). Animals do not usually follow a stimulus amplitude waveform precisely and the task is also trivial in the sense of signal processing. The AM representations in the middle to upper layers were to some degree similar to those of the middle brain regions, but no layers exhibited an AM representation similar to that of the central brain regions (Fig. 12*a*,*b*, red crosses). In the upper layers, only small numbers of rMTFs exhibited clear tuning and the tBMFs and tUCFs were higher than those of the central brain regions (Fig. 12*a*, right column). The result indicates that the emergence of auditory-system-like AM tuning in the upper layers requires natural objectives and the waveform-following task did not induce such a representation even if the input data consisted of natural sounds.

Together, the modification of the category labels, the sound statistics, and the optimization objective caused the auditory-system-like AM representations in the middle layers and above to deteriorate. Lower layers never exhibited AM tuning consistently across all conditions, probably because of the nature of the cascading architecture. The middle layers exhibited auditory-system-like AM tuning when trained on natural input sounds and the proper sound–category correspondence. The upper layers exhibited auditory-system-like AM tuning when optimized for the categorization task but not for the waveform following task (Table 2).

**Table 2.** Major factors for AM representation in different regions

Regions	Major factor
Higher	Optimization objective
Middle	Data naturalness
Lower	Cascading architecture

### Generality across datasets

**Figure 13.**
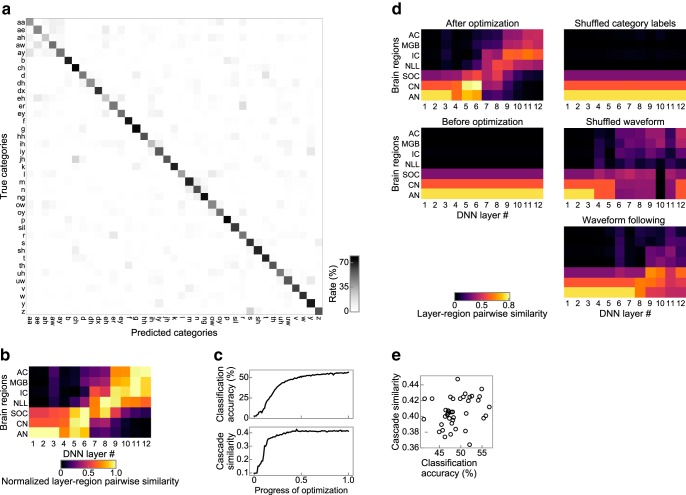
Similarity emerges consistently from speech dataset. ***a***, Confusion matrices of the classification of the validation data. There are 39 categories. Other conventions are the same as in Figure 3. ***b***, Layer–region pairwise similarity normalized by the maximum value for each brain region. Other conventions are the same as in Figure 8*c* ***c***, Classification accuracy (top) and cascade similarity (bottom) as functions of the progress of optimization. ***d***, Layer–region pairwise similarity after and before optimization, that of the DNN trained on shuffled category labels and shuffled waveforms, and that of the waveform-following task. ***e***, Cascade similarities of DNNs with various architectures plotted against their classification accuracies. All results were consistent with those obtained with the nonhuman natural sound.

As a DNN trained on one dataset recognizes another dataset very well with only a slight modification (Yosinski et al., 2014), it may be possible that AM tuning can also be generalized across datasets. Previous studies provide positive pieces of evidence: a machine learning model trained for substantially different sound datasets has exhibited a similar representation of AF (Smith and Lewicki, 2006). To test the generality of the finding that we report in the present study across datasets, we conducted neurophysiology in a DNN optimized for the recognition of vocal elements in speech (Fig. 13*a*).

The speech dataset provided essentially the same conclusions as those obtained with animal and environmental sounds. The layer–region pairwise similarity matrix exhibited a diagonal pattern (Fig. 13*b*). The lower, middle, and upper layers were similar to the peripheral, middle, and central regions, respectively. The similarity emerged during the optimization (Fig. 13*c*; Spearman's rank correlation coefficient = 0.83, *p* = 3.76 × 10^−27^, *n* = 100) and was weak under control conditions (Fig. 13*d*). The similarities in the DNNs with various architectures correlated with the classification accuracy (Fig. 13*e*; ρ = 0.33, *p* = 3.91 × 10^−2^, *n* = 39). The results indicate that auditory-system-like AM tuning emerged robustly across different datasets.

### Sharpness of the tMTF

**Figure 14.**
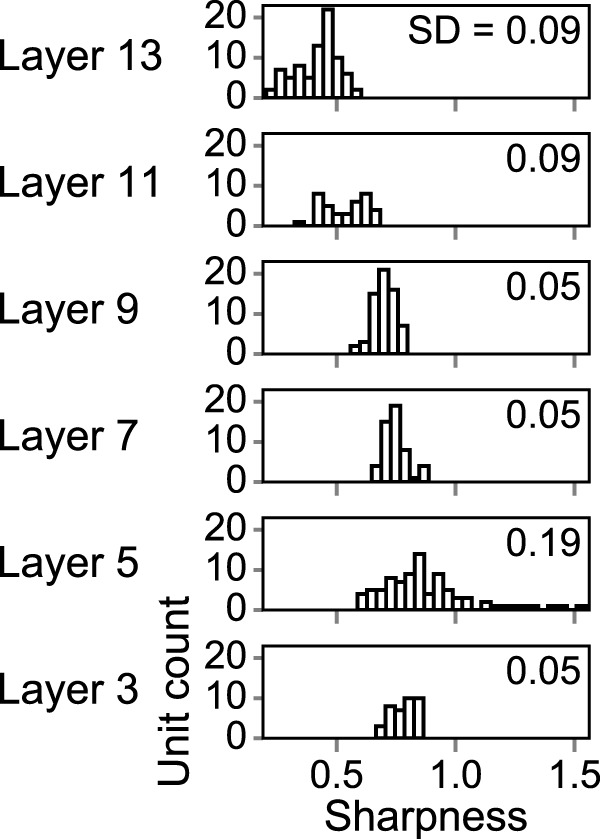
Histograms of tMTF sharpness. Layers 3, 5, 7, 9, 11, and 13 are shown as examples. The Q factors in the first and second layers are not calculated because no units in these layers were band-pass shaped. SDs are shown in the top right corners.

Thus far, we have focused on the BMF and UCF for characterizing the MTF. Another aspect often considered is the sharpness of a tMTF, which is represented by quality factors (Q factors). Here, we calculated the Q factors of the unit tMTFs as in a previous physiological study (Rodríguez et al., 2010). The distribution of Q factors appeared different in different layers and, within a layer, they were confined to a narrow range (Fig. 14). This is demonstrated by the small standard deviations of the distributions (shown on the right in the histograms). Also, in most layers, the Q factors were <1. These results indicate the emergence of broadly tuned tMTFs with a relatively constant sharpness, which is consistent with the Q values in animals reported in previous studies (Ewert and Dau, 2000; Lorenzi et al., 2001; Rodríguez et al., 2010).

### Tuning to acoustic frequency

**Figure 15.**
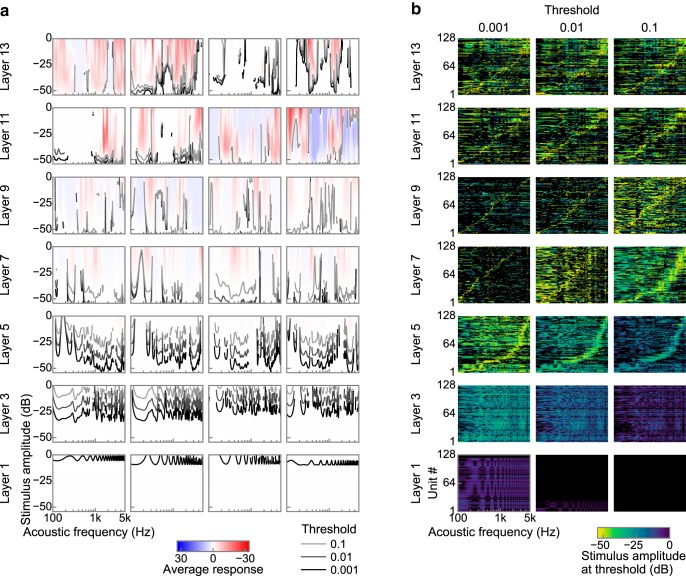
Tuning to acoustic frequency. ***a***, AF tuning in four example units in each layer. Red and blue, respectively, indicate larger and smaller responses than the silent stimulus. White indicates a response equal to silence. Black and gray lines show the AF tuning curves. The thresholds were 0.1 (light gray lines), 0.01 (dark gray lines), and 0.001 (black lines) above the response to silence. Generally, the responses appeared monotonic along the stimulus amplitude, but some units in the upper layers exhibited a nonmonotonic response along the stimulus amplitude. The AF tuning curves did not show clear single troughs. ***b***, AF tuning curves in all the units in each layer. The curves are shown for thresholds of 0.001 (left), 0.01 (middle), and 0.1 (right) above the response to silence. The units in each layer are sorted by the trough frequency of the tuning curves. Troughs in the AF tuning curves in the middle layers appear to cover a wide AF range, but not in the lower and higher layers.

We also examined tuning to the AF, which is the most frequently measured characteristic in auditory science (Pickles, 2012). We presented sinusoids with various AFs and amplitudes to the DNN and characterized the single-unit responses with the temporal average of the units' activities (Fig. 15*a*). The responses generally increased as the input amplitude increased, but some units in the upper layers exhibited nonmonotonic responses to the input amplitude. As in the neurophysiological studies, a unit was characterized by an AF tuning curve, namely the minimum stimulus amplitude that provides a response larger above a certain threshold (Fig. 15*a*, gray and black lines, 15*b*). Tuning curves from the first to third layers exhibited many troughs (or local minima). Those around the fifth layer exhibited a small number of major troughs and many minor troughs. The major trough of a unit can be interpreted as exhibiting a band-pass property. The center frequencies of the major troughs of the unit population spanned a wide AF range (Fig. 15*b*), which may be interpreted as a band-pass filter bank. The tuning curves in higher layers were more complex without clear band-pass-like tunings. The overall results were in contrast to those for the auditory system, where neurons across many regions usually exhibit AF tuning with a relatively sharp single peak. This property is likely to originate from AF decomposition occurring in the cochlea. We did not explicitly implement the spectral decomposition of the input sound but directly fed raw waveforms to the DNN. The results suggest that AF decomposition in the cochlea may be essential for auditory-system-like AF tuning, but not for auditory-system-like AM tuning.

## Discussion

We found that a DNN optimized for natural sound recognition exhibits an AM tuning similar to that of the auditory system throughout the entire cascade of layers. Because our DNN was not designed or trained to reproduce any physiological or anatomical properties of the auditory system, the results should reflect only the nature of the task and the data. Therefore, AM tuning in the auditory system might also emerge during evolution and development via optimization to sound recognition in the real world.

### Physiology in a DNN

Although DNNs have explained sensory representation in several modalities (Khaligh-Razavi and Kriegeskorte, 2014; Yamins et al., 2014; Horikawa and Kamitani, 2017; Zhuang et al., 2017; Cueva and Wei, 2018; Kell et al., 2018), to the best of our knowledge, this is the first report of the similarity throughout the entire cascade of sensory processing. This could be realized by single-unit recording, which is a highly general neurophysiological technique, on a DNN performing sound recognition from a raw sound waveform. The generality of single-unit recording enabled us to take advantage of the long-accumulated neurophysiological knowledge of AM representation in a variety of brain regions and modeling the entire auditory process enabled us to map all the stages of the process to the corresponding brain regions. Although this study focused on AM representation, the neural representation of any domains of stimulus parameters can be explored using the same paradigm as long as the property of interest can be measured with single-unit recording.

From the perspective of machine learning, our results suggest the effectiveness of analyzing DNNs with physiological methods. To date, various methods have been proposed for analyzing representation in a DNN (Montavon et al., 2018). Most of them rely on the differentiability of the DNN and use backpropagation to estimate the optimal input for each unit assuming such an input exists. By contrast, there is a long history regarding the development of a physiological method for explaining biological neurons, to which backpropagation cannot be applied (Sharpee et al., 2011). The success of our paradigm opens up the future possibility of using well established physiological methods to explore the stimulus representation of a DNN and other complex machine learning models.

### AM representation in different regions

The result showing that there was little tuning in the peripheral region may be due to the architecture in which simple operations are cascaded. Computing the AM rate requires at least envelope extraction and the frequency decomposition of the envelope. A small number of peripheral brain regions are probably incapable of such nontrivial computation.

Midlevel neural processing may be a necessary step if the brain is to form a proper stimulus representation for further processes in various tasks. Because lower layers do not tune to an AM rate, the middle layers are effectively the first layers that process AM signals. It is reasonable to think that the first stage of the data process is affected by the data structure and is critical for later recognition.

A higher representation may be directly used for the final recognition process. In other words, whatever the stimulus representation is, the role of the central auditory regions is to derive appropriate outputs for the specific task.

### Reduction in temporal resolution

Both of the prominent characteristics of auditory AM coding, namely a decrease in synchronizing AM rate and time-to-rate conversion, involve a reduction in a signal's temporal resolution. Why is such a scheme beneficial for sound recognition? With our model, as in a typical recognition task with a DNN, the final output at each time frame is the category label assigned to the unit with the maximum activation at the time in the classification layer (the layer above the 13th layer). If the units synchronize with a fast AM, then the output category will be temporally unstable. Conversely, if the activation of a classification unit is large all the time, then the DNN will output a constant category over time. The latter case is preferable for the classification of sounds of a reasonable duration.

Strictly speaking, by avoiding the pooling operation that is often included in typical DNNs, the layers in our DNN do not necessarily down-sample the input. However, even without pooling, it is still possible that successive integration by convolution and half-wave rectification could bias the DNN toward implicitly extracting relatively clean temporal properties such as envelopes. From the result of the waveform following experiment (Fig. 13*d*), at least we can conclude that proper setting for optimization is necessary for down-sampling properties to emerge in the DNN. Although we avoided making explicit assumptions on DNN parameters by learning them for sound recognition, the architecture of the DNNs with cascaded nonlinearity could induce some significant biases regarding the temporal properties of the extracted signals. Future experiments without half-wave rectification or convolution may reveal the effect of these operations.

### Sharpness of a tMTF

In neurophysiological and psychophysical studies, tMTFs have also been characterized by their sharpness. One study has shown that most of the neurons recorded in the central nucleus of the IC exhibit Q factors between 0.5 and 1.5 (see supplemental figure S3*A* in Rodríguez et al., 2010). Interestingly, layers 7–9 in our DNN, which exhibited IC-like sharpness distributions, were also similar to the IC in terms of BMF/UCF distributions (Fig. 8*c*). Also, although unitwise Q factors may not be directly comparable to those of psychophysical tMTFs, most Q values in our DNN were <1 and thus fall in the range suggested by psychophysical studies (Ewert and Dau, 2000; Lorenzi et al., 2001). These results suggest that the broad tuning to the AM rate, which is seen in the auditory system, may be effective for natural sound recognition.

### AF tuning

Unlike neurons in the auditory system, our DNN did not exhibit sharp single troughs in the AF tuning curves, although some other studies have reported auditory-like AF tuning emerging in a DNN with a different architecture from ours (Hoshen et al., 2015; Terashima and Furukawa, 2018). In the auditory system, the AF tuning of a neuron depends largely on the mechanical and physical properties of the cochlea (Pickles, 2012). Some architectural constraints might be necessary to induce similarity in the auditory system in the AF domain. By not using a spectrogram as an input, the use of the spectral information such as harmonics for sound recognition may become more direct, which may lead to the complex spectral selectivity shown in our AF tuning curves. The application of a similar convolutional DNN to the temporal and spectral dimensions of a spectrogram might result in more organized “spectrotemporal” tunings similar to those in the auditory system, although an investigation of what determines the shape of an AF tuning curve in a DNN is beyond the scope of this study.

Several other computational studies have tried to explain auditory AM coding by using models with anatomical and physiological assumptions, including AF decomposition in a cochlea (Pešán et al., 2015; McWalter and Dau, 2017; Khatami and Escabi, 2018). In contrast, our results indicate that auditory-like AM coding emerges even without cochlear AF decomposition. Sharp AF tunings are probably unnecessary to obtain an effective AM representation for natural sound recognition.

### Potential impact on plasticity studies

In this study, we analogized the auditory system with the optimized DNN. It is difficult to clearly identify the biological counterparts of the DNN optimization process. They probably include a mixture of the effects of short-term plasticity and long-time evolution over generations. Although it may be impractical to experimentally manipulate the long-time evolution in humans, studies with various AM detection or discrimination tasks in humans suggest that the responses of central auditory neurons to AM cues are plastic and that practice may modify the AM processing circuitry (Fitzgerald and Wright, 2005, 2011; Rosen et al., 2012; Sabin et al., 2012; Caras and Sanes, 2015; Joosten et al., 2016). It may be interesting future work to use a DNN model like ours to explore the mechanisms underlying such short-term plasticity.
